# Postconditioning with simvastatin decreases myocardial injury in rats following acute myocardial ischemia

**DOI:** 10.3892/etm.2015.2273

**Published:** 2015-02-06

**Authors:** HENG-CHEN YAO, LAN-JU YANG, QIAN-FENG HAN, LAN-HUA WANG, LEI WU, CHUN-YAN ZHANG, KE-LI TIAN, MEI ZHANG

**Affiliations:** 1 Department of Cardiology, Qilu Hospital of Shandong University School of Medicine, Jinan, Shandong 250012, P.R. China; 2 Department of Cardiology, Liaocheng People’s Hospital of Taishan Medical University, Liaocheng, Shandong 252000, P.R. China; 3 Department of Biochemistry and Molecular Biology, ShandongUniversity School of Medicine, Jinan, Shandong 250012, P.R. China

**Keywords:** simvastatin, acute myocardial infarction, postconditioning, high mobility group box 1

## Abstract

The aim of the present study was to investigate whether postconditioning with simvastatin attenuated myocardial ischemia reperfusion injury by inhibiting the expression of high mobility group box 1 (HMGB1) in rat myocardium following acute myocardial ischemia. In total, 30 male Sprague-Dawley rats were divided into sham operation (sham; n=10), acute myocardial infarction (AMI; n=10) and simvastatin (sim; n=10) groups. The AMI and sim groups were subjected to ischemia for 30 min, followed by reperfusion for 180 min. The rats in the sim group were administered 20 mg/kg simvastatin intravenously 5 min prior to reperfusion. Subsequently, the infarct size, serum cardiac troponin (c-TnI), tumor necrosis factor (TNF)-α and myocardial malondialdehyde (MDA) levels and superoxide dismutase (SOD) activity were measured. Western blot analysis was used to detect the protein expression of HMGB1. Postconditioning with simvastatin was shown to decrease the infarct size and HMGB1 expression levels in the myocardium following AMI (P<0.05). In addition, postconditioning with simvastatin not only decreased the serum levels of c-TnI and TNF-α (P<0.05), but also inhibited the increase in MDA levels and the reduction in SOD activity (P<0.05). Therefore, postconditioning with simvastatin was shown to attenuate myocardial injury. The underlying mechanism may be associated with the downregulation of HMGB1 expression in the ischemic myocardium.

## Introduction

High mobility group box 1 (HMGB1) is a ubiquitous and abundant nuclear protein. HMGB1 can be passively released into the extracellular space in response to necrotic signals or actively secreted in response to inflammatory signals (1,2). Previous studies have shown that HMGB1 functions as a proinflammatory cytokine in certain cardiovascular diseases, and is associated with the severity of coronary artery disease (3,4).

Statins (3-hydroxy 3-methylglutaryl coenzyme A reductase inhibitors) are a class of cholesterol-lowering drug that are widely used in the prevention and treatment of cardiovascular diseases (5). Previous studies have shown that preconditioning with statins may attenuate myocardial injury in rats following acute myocardial infarction (AMI) (6,7).

In cases of acute myocardial infarction, rapid reperfusion by percutaneous coronary intervention or thrombolytic therapy is important to salvage myocardial tissue from necrosis and reduce the size of infarct tissue. Paradoxically, reperfusion is frequently associated with an exacerbation of tissue injury, enlargement of the infarct size, and a marked inflammatory response, known as ischemia/reperfusion injury (8). Ischemia/reperfusion injury is defined as the necrosis of those cells that remained viable following a period of ischemia.

Postconditioning is defined as the rapid sequential intermittent interruption of blood flow applied during early moments of reperfusion. Postconditioning has been demonstrated to attenuate organ injury, including the heart (9), spinal cord (10), brain (11), kidneys (12), liver (13), muscle (14), lungs (15) and intestines (16) in previous studies. Drug postconditioning involves the administration of a protective substance immediately prior to reperfusion and may be performed as a post-ischemic intervention to reduce myocardial tissue damage during the study of ischemia/reperfusion injury. There are a number of controversial issues regarding the efficacy of postconditioning in various animal models with comorbidities; however, the majority of preclinical studies have indicated that postconditioning is an effective intervention for reducing organ necrosis and apoptosis. Ischemia postconditioning may be able to reduce ischemia/reperfusion injury in cases of myocardial infarction by inhibiting the inflammatory response and reducing the levels of tumour necrosis factor (TNF)-α and interleukin-6 (17).

Simvastatin is a type of HMG-CoA reductase inhibitors. As simvastatin is inexpensive, it has been widely used in the treatment of coronary heart disease and associated conditions in China.

However, whether postconditioning with simvastatin is able to alleviate myocardial injury is yet to be fully understood. Thus, the present study investigated the effect of simvastatin postconditioning on myocardial ischemia reperfusion injury, and the mechanism underlying the protective effect.

## Materials and methods

### Animal model

The study was approved by the Institutional Review Board of Liaocheng People’s Hospital of Shandong Province (Liaocheng, China). In total, 30 male Sprague-Dawley rats (weight, 250–300 g) were obtained from the Experimental Laboratory of Shandong University of Traditional Chinese Medicine (Jinan, China). The rats were housed in a quiet environment with a humidity of 60±5% and a temperature of 22±2°C. A 12-h light-dark cycle (light beginning at 8am) was applied, with all operations performed during the light phase of the cycle. The rats were divided at random into three groups that each received a different treatment. The sham operation group (sham; n=10) rats underwent surgery without the ligation of the left anterior descending artery (LAD). AMI group (n=10) rats were subjected to LAD occlusion for 30 min, followed by reperfusion for 180 min. The simvastatin group (sim; n=10) rats were subjected to LAD occlusion for 30 min, followed by reperfusion for 180 min. Furthermore, 20 mg/kg simvastatin was dissolved in saline and injected intravenously at <5 min prior to reperfusion (6). Rats in the sham and AMI groups received equal volumes of normal saline at the same time points.

A rat model of AMI was established in accordance with previously reported methods (7,18). The rats were anesthetized with an intraperitoneal injection of 60 mg/kg sodium pentobarbital and the trachea was cannulated for artificial ventilation with room air at a rate of 55 breaths/min. The inspiratory/expiratory ratio was set to 1:1 and the tidal volume was adjusted to 2–3 ml/100 g body weight. The body temperature of the rats was maintained at 37±0.5°C with a heating pad. Lead II of an electrocardiogram (ECG) was monitored with stainless needle electrodes that were attached to the rat limbs. The ECG was recorded and analyzed using an ECG-6511 data acquisition system (Guangdian Medical Devices Co., Ltd., Shanghai, China).

An incision was made by a left thoracotomy through the fourth intercostal space. Following a pericardiotomy, a 5-0 silk ligature attached to a small needle was placed around the left coronary artery close to its origin, and complete occlusion of the coronary artery was verified by ST segment elevation on the body surface ECG. In the sham operation group, the needle and ligature were placed at the origin of the left coronary artery; however, complete ligation was not conducted. Prior to ligation of the LAD, 200 IU/kg heparin was intravenously administered.

### Biochemical analysis

Following reperfusion for 180 min, blood samples were obtained from the right femoral vein. After leaving to settle for 30 min, the blood samples were centrifuged at 1,400 × g for 10 min. The serum samples were stored at −80°C for subsequent analysis. Serum levels of TNF-α and cardiac troponin (c-TnI) were measured using previously reported methods (7).

Following reperfusion for 180 min, the rat hearts were removed and washed with normal saline. A 0.5-g sample of ischemic heart tissue was obtained and ground to powder at 0–4°C. Next, the myocardial homogenate was centrifuged at 2,000 × g for 30 min. The supernatant was collected and stored at −80°C until required for the MDA concentration and SOD activity assays. An MDA assay kit and SOD assay kit (Nanjing Jiancheng Bioengineering, Co. Ltd., Nanjing, China) were used to measure the MDA concentration and SOD activity, following the manufacturer’s instructions.

### Assessment of infarct size

Infarct size was assessed by 2,3,5-triphenyltetrazolium chloride (TTC) staining, using a previously reported method (7). Following reperfusion for 180 min, the LAD was reoccluded and 1 ml Evans blue dye (2.0%) was injected via the femoral vein. The entire heart was excised, rinsed of excess Evans blue dye and the right ventricle and right and left atria were removed. The remaining left ventricle was frozen at −80°C. The frozen left ventricle was sliced horizontally from the apex to the base, yielding five slices. The slices were incubated in 1% TTC for 15 min at 37°C. The viable myocardium was stained red and the infarcted myocardium was stained white. The slices were photographed using a PowerShot N digital camera (Canon, Inc., Tokyo, Japan). The borders of the infarct, ischemic and nonischemic areas of the heart images were traced and measured using Image-Pro Plus 3.0 (Media Cybernetics, Inc., Rockville, MD, USA). Infarct sizes were expressed as a percentage of the risk area volume (infarct size/risk area).

### Western blot analysis of HMGB1 expression levels

Myocardial HMGB1 protein expression levels were measured using western blot analysis, following previously reported methods (7). Myocardial proteins were extracted using a lysis buffer containing 20 mM Tris (pH 7.5), 150 mM NaCl, 1 mM EDTA, 1 mM EGTA, 1% Triton X-100, 2.5 mM sodium pyrophosphate, 1 mM β-glycerophosphate, 1mM Na_3_VO_4_ and 1 μg/ml leupeptin. Phenylmethylsulfonyl fluoride (1 mM) was added immediately prior to use. Protein concentrations were determined using Pierce bicinchoninic acid protein assay kit (#23225; Pierce Biotechnology Inc., Rockford, IL, USA), and 50 μg protein was loaded onto and separated by SDS-PAGE and subsequently transferred onto a polyvinylidene fluoride membrane. After blocking for 30 min with 5% skimmed milk, the membranes were incubated with a monoclonal rabbit anti-HMGB1 primary antibody (1/30,000, #ab79823; Abcam, Cambridge, UK) for 1 h, washed and incubated for 30 min with a goat anti-rabbit IgG (H+L) horseradish peroxidase-conjugated secondary antibody (1:1,000, #A0208; Beyotime Institute of Biotechnology, Haimen, China). Bands were detected with with BeyoECL Plus enhanced chemiluminescence reagent (#P0018; Beyotime Institute of Biotechnology). β-Actin (Abcam) was detected as a loading control.

### Statistical analysis

Data are expressed as the mean ± standard deviation or as percentages where appropriate. SAS 6.12 software (SAS Institute, Inc., Cary, NC, USA) was used for statistical processing. One-way analysis of variance was used to analyze the mean values between groups, where P<0.05 was considered to indicate a statistically significant difference.

## Results

### Serum levels of c-TnI and TNF-α

Serum levels of c-TnI and TNF-α in the sim group decreased when compared with the AMI group (P<0.05; Table I). In addition, serum levels of c-TnI and TNF-α in the sham group were lower when compared with the AMI and sim groups (P<0.05).

### MDA levels and SOD activity

Following reperfusion for 180 min, the MDA levels in the AMI group increased significantly, while the SOD activity levels decreased significantly, when compared with the sham group (P<0.05). The increase in MDA levels and reduction in SOD activity were significantly inhibited by simvastatin treatment (P<0.05; Table I).

### Comparison of infarct size between the groups

Infarct sizes in the sim group rats were significantly decreased when compared with the AMI group rats (P<0.05).

### Protein expression levels of HMGB1

**Figure 1:**
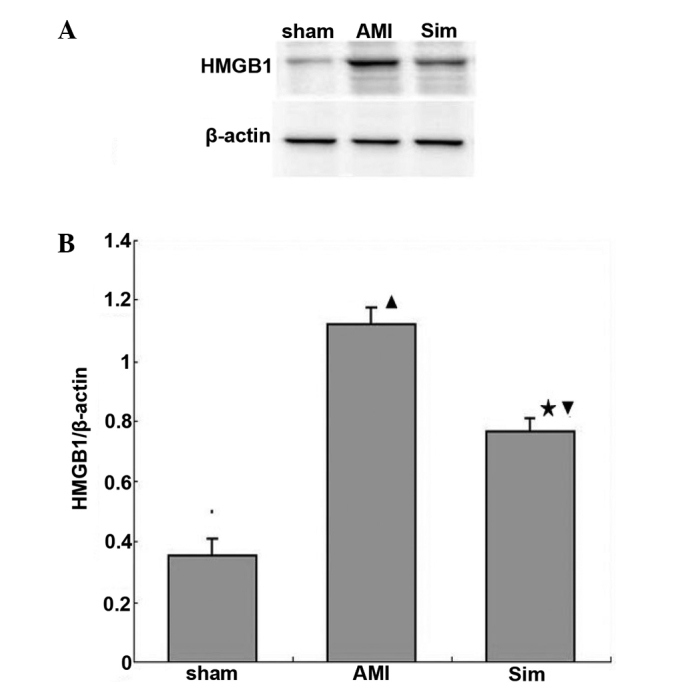
(A) HMGB1 expression levels in the ischemic areas of the left ventricle samples were examined by western blot analysis, with the expression of HMGB1 protein normalized against β-actin expression. (B) Densitometric analysis of the expression levels following normalization against β-actin. The myocardial expression of HMGB1 in the sim group decreased significantly when compared with the AMI control group. ^▲^P<0.01 and ^★^P<0.05, vs. sham; ^▼^P<0.05, vs. AMI. HMGB1, high mobility group box 1; Sham, sham operation; AMI, acute myocardial infarction; Sim, simvastatin.

Expression levels of HMGB1 protein in the myocardium decreased in the sim group when compared with the AMI group (P<0.05; Fig. 1).

## Discussion

The present study demonstrated that myocardial expression of HMGB1 significantly increases in rats with AMI and postconditioning with simvastatin reduces the infarct size, exerting an anti-inflammatory effect and decreasing the myocardial expression of HMGB1. These results indicated that simvastatin postconditioning may alleviate myocardial injury following acute myocardial ischemia by reducing the expression of HMGB1.

Reperfusion of the ischemic myocardium is important for protecting myocardial tissue against necrosis following acute myocardial ischemia. However, the premature opening of an occluded coronary artery may result in myocardial ischemia/reperfusion injury (19). Therefore, the alleviation of myocardial ischemia/reperfusion injury is an important approach for the management of acute myocardial ischemia.

TNF-α strongly induces apoptosis and necrosis in myocytes and is known to stimulate the remodeling process and provoke myocardial dysfunction following AMI (20). C-TnI, which may be released in infarct areas of the myocardium, is often elevated in cases of AMI. Previous studies have revealed that TNF-α and c-TnI are significantly elevated in AMI patients (4,7). HMGB1 is actively secreted by macrophages and monocytes, or released by necrotic cells into the extracellular milieu, where the protein may trigger inflammation (21). A previous study showed that HMGB1 was able to stimulate the production of TNF-α (22).

In accordance with these previous studies, the present study demonstrated that the ischemia-induced upregulation of TNF-α was inhibited by postconditioning with simvastatin. Furthermore, the myocardial expression of HMGB1 was decreased by postconditioning with simvastatin. Therefore, it is feasible that simvastatin, which is able to substantially reduce the expression of proinflammatory factors, may exert a cardioprotective effect by decreasing the myocardial expression of HMGB1.

During the process of ischemia/reperfusion injury, a cascade of inflammatory responses are initiated, including oxidative stress generation and cytokine production (23). Proinflammatory cytokines may further promote inflammatory cell adhesion and infiltration into the ischemic myocardium and enhance acute tissue injury (24). Previous studies have shown that hypoxic hepatocytes release HMGB1 through an active process facilitated by the production of radical oxygen species (ROS). ROS, in turn, induce the release of HMGB1. Antioxidants are able to reduce the release of HMGB1 and liver damage in liver ischemia/reperfusion injury (25,26). MDA levels are often used as an index of ROS, while SOD activity is used as an indicator of lipid superoxide levels in the ischemic myocardium.

In the present study, the MDA levels in the AMI group were increased significantly and the SOD activity levels were significantly decreased when compared with the sham group. The increase in MDA levels and reduction in SOD activity were significantly inhibited by simvastatin postconditioning. Furthermore, the myocardial expression of HMGB1 and the infarct size in the sim group were significantly reduced when compared with the AMI group. These results indicated that simvastatin may exert a cardioprotective effect by decreasing the myocardial expression of HMGB1, which may be associated with the myocardial ischemia-induced inhibition of ROS. This conclusion was consistent with the results of a recent study (27).

The exact mechanism by which simvastatin protects the heart against myocardial injury caused by acute myocardial ischemia remains unclear. A prior study showed that when Toll-like receptor 4 was mutated or deficient, the rate of cardiomyocyte apoptosis and the infarct size decreased, with the expression of HMGB1 and TNF-α notably inhibited following myocardial ischemia/reperfusion (28). Furthermore, it has been reported that the cardioprotective effects of simvastatin against infarction are associated with a reduction in myocardial edema. Simvastatin exerts this effect on edema by suppressing the expression of aquaporin-1, -4, -8 and -9 in a partially protein kinase A-dependent manner (29). However, the underlying mechanisms of the infarct size-limiting and cardioprotective effects of simvastatin are not yet fully understood.

The present study was consistent with previous studies in finding that postconditioning with simvastatin may attenuate myocardial injury induced by acute myocardial ischemia. In particular, the present study showed that this effect may be associated with the downregulation of HMGB1 expression in the myocardium.

In conclusion, postconditioning with simvastatin inhibited the myocardial expression of HMGB1, which may be associated with its cardioprotective effects.

## Figures and Tables

**Table I:** Values of c-TnI, TNF-α, SOD, MDA and IS.

Variables	Sham (n=10)	AMI (n=10)	Sim (n=10)
c-TnI (ng/ml)	0.96±0.34	14.21±9.24^a^	7.42±4.06^a^^b^
TNF-α (pg/ml)	0.75±0.21	1.76±0.33^a^	1.14±0.24^a^^b^
SOD (μU/l)	146.52±14.37	95.76±12.45^a^	112.05±13.42^a^^b^
MDA (μmol/l)	1.81±0.83	5.11±1.17^a^	3.43±1.41^a^^b^
IS (%)	-	56.38±8.58	24.37±4.2^b^

Data are presented as the mean ± standard deviation.

a P<0.05, vs. sham group;

b P<0.05, vs. AMI group.

AMI, acute myocardial infarction; Sim, simvastatin; c-TnI, cardiac troponin; TNF-α, tumor necrosis factor-α; SOD, superoxide dismutase; MDA, malondialdehyde; IS, infarct size.
